# Synthesis and Characterization of Thymol-Loaded Niosomal Film for the Prevention of Implant-Related Infection

**DOI:** 10.52547/ibj.3788

**Published:** 2023-02-25

**Authors:** Raziyeh Najafloo, Rana Imani, Mahla Behyari, Shirin Nour

**Affiliations:** Department of Biomedical Engineering, Amirkabir University of Technology (Tehran Polytechnic), Tehran159163-4311, Iran

**Keywords:** Thymol, Infections, Wound healing

## Abstract

**Background:**

Infection is one of the significant challenges in medical implant-related surgeries. Despite systemic antibiotic therapies, bacterial growth after implantation may cause implant failure. Nowadays, unlike the systemic therapy, local controlled release of antibiotic agents is considered an effective approach for the prevention of implant-related infections. The present study aimed to develop a niosomal nanocarrier incorporated into fibroin films for local and continuous delivery of thymol, a natural plant-derived antimicrobial agent for preventing infections caused by implant-related.

**Methods:**

Niosomes containing thymol were prepared by thin-film hydration technique. Thymol sustained release from the prepared films was assessed for 14 days. Antibacterial activities of the synthesized films were also evaluated by the agar diffusion technique against *Escherichia coli*,* Pseudomonas aeruginosa*, and* Staphylococcus aureus.*

**Results:**

The release behavior from the niosomal thymol films showed a sustained manner, in which the amount of the released thymol reached 40% after 14 days. The films containing thymol with and without niosome showed a significant viability against L929 fibroblast cells compared to other groups after 24 and 48 h, using MTT assay. Also, samples exhibited potent antibacterial activity against Gram-negative and Gram-positive bacteria.

**Conclusion:**

The results of this study demonstrate that the niosomal thymol-loaded fibroin film is a promising candidate for the controlled release of thymol and prevention of implant-related infection.

## INTRODUCTION

One of the main challenges in the treatment of patients underwent implantation is the probable occurrence of infection and abscess formation, which may lead to implant rejection^[^^1^^]^. The reasons for this response are the formation of bacterial biofilm and immune reaction against the implant^[^^2^^]^. Despite improvements in systemic antibiotic therapy, lack of blood supply in implantation sites results in insufficient concentration of antibiotic agent in these regions, and accordingly bacterial resistance can happen^[^^3^^]^. Compared to the systemic administration, the local administration of the antibacterial agents has been recommended for the treatment and prevention of infection in biomaterial implantation sites^[^^4^^]^. Studies have suggested the efficiency of using plant-derived antibacterial agents in treating infections^[^^5^^]^. The extracts derived from plant often have low risk of bacterial resistance^[^^6^^]^. 

Thymol, known as 2-isopropyl-5-methyl phenol (IPMP), is a natural monotrepene phenol derivative of Thymus and Origanum plants^[^^7^^]^. Thymol showed strong antimicrobial properties against Gram-positive bacteria, Gram-negative bacteria, fungi, and yeasts^[^^8^^]^. Many researchers have indicated the benefits of thymol in the medical, agricultural, food, and pest control fields^[^^9^^-^^11^^]^. The antibacterial mechanism of thymol relies on the disruption of the cytoplasmic membrane of microorganisms. In other words, thymol, due to the lipophilic properties and low solubility, was expected to interact with phospholipidic membranes and cause disturbance in lipid membranes^[^^5^^,^^8^^]^. A major challange related to thymol and other plant extracts is their low water solubility and stability, which may reduce their antibacterial activity. Accordingly, encapsulation of these compounds into appropriate nanocarrier can increase their stability, and subsequently optimize their release profile^[^^12^^-^^15^^]^. Niosomes are non-ionic surfactant vesicles that could be used to deliver both hydrophilic and hydrophobic medicinal substances^[^^16^^-^^19^^]^. Niosomes are biodegradable, biocompatible and non-toxic carriers and have high efficiency to encapsulate drugs in the small volume of vesicle^[^^20^^]^. Therefore, niosomes have been considered as proper drug carriers for delivery of the antibiotic agents, because they are able to maintain the bioactivity of the loaded agents and provide the sustained release platform^[^^21^^-^^23^^]^. Owing to biocompatibility, mechanical stability, controllable degradability, and acceleration effect on wound healing process, SF is utilized as a favorable biomaterial in various applications, including wound dressings^[^^24^^]^, tissue engineering scaffold^[^^25^^,^^26^^]^, and drug delivery systems^[^^27^^]^. 

In this study, thymol-loaded niosomes were fabricated via thin-film hydration method and then incorporated into fibroin films. The physicochemical characteristics and release behavior of nanocarriers and films were evaluated precisely. Also, the antibacterial characteristics of the produced films containing thymol-loaded niosome were investigated using the disk diffusion method against *Pseudomonas aeruginosa, Escherichia coli*, and *Staphylococcus aureus* bacteria.

## MATERIALS AND METHODS

**Preparation of thymol-loaded niosomes**

Niosomes containing thymol were prepared by thin-film hydration technique^[^^28^^]^. Briefly, Span 60 (Sigma, USA), cholesterol (Sigma), and thymol (Merck, Germany) with the 3:2:0.1 weight ratio were dissolved in 15 ml of ethanol. The organic solvent was evaporated by a rotary evaporator (Heidolph, Germany) at 150 rpm at 40 °C. The obtained film was hydrated with 30 ml of PBS. Thereafter, an extensive vortexing and sonication were performed in an ultrasonic bath (CD-4820-Digital Ultrasonic Cleaner, China) for 45 min and 24 min, respectively.

**Preparation of niosomal SF film**

Raw SF was degummed twice with 0.5% (W/W) sodium carbonate (Merck) solution at 100 °C for 30 min. Then the degummed SF was washed with warm distilled water and dissolved in a 9.4 M lithium bromide (Merck) solution at 60 °C for 4 h. The cellulose tubular membrane was used to do dialysis in distilled water for three days, and then the prepared SF solution was freeze-dried to achieve SF sponges. Next, we prepared three film samples of SF, including SF film, SF film containing niosomal thymol (SF/N/Th), and SF film containing thymol (SF/Th). Each sample was produced by stirring 0.25 g of SF, 2.5 ml of formic acid, and 0.5 ml of niosome suspension (or 0.5 mg of thymol for SF/Th fabrication) for 20 minutes, followed by sonication for 30 minutes. Finally, the prepared degassed solutions were spread over a glass plate and dried at room temperature for 4 days to obtain film samples. 

**Characterization of niosomes and niosomal film**

***SEM and DLS***

For SEM analysis, the niosomal suspension was diluted and dried using a freeze dryer. After spin coating with gold, the morphology and shape of the niosomes were observed by a SEM microscope (Phenom Prox, The Netherlands) under 25 KV. The hydrodynamic size of niosome and distribution of the size were determined by DLS (Malvern instruments Ltd., Worcestershire, UK) at a fixed scattering angle of 135° and 25°C. Polydispersity index (a measure of homogeneity) and apparent z-average hydrodynamic radius were then achieved. In this experiment, the average value of three samples was reported.

***EE of thymol***

Before the analysis of %EE, the UV-visible absorption at the wavelength of 279 nm against different specified concentrations of thymol )0, 16, 24, 33, 41, and 50 µg/ml) was plotted as a standard curve. To evaluate the %EE of thymol, 1.5 ml of the niosomal suspension was centrifuged at 15,000 rpm for 10 minutes to separate supernatant. Then the absorbance of the supernatant at the wavelength of 279 nm was determined by a UV-visible spectrophotometer (Varian-Inc, USA) to measure the amount of the unloaded thymol using the prepared absorption-concentration standard curve. %EE was estimated by the following equation:

Thymol encapsulation efficiency (%) = (W_t_-W_f_) × 100 /W_t _ (1)

Where W_t_ is the initial amount of thymol in the solution of niosome, and W_f_ shows the amount of the un-loaded thymol in the supernatant.

***In vitro release study***

Thymol release profile from niosomes in the in vitro conditions was evaluated using the dialysis method. Briefly, 10 ml of PBS was added to 3.31 g of the dried niosomes, and the obtained suspension was poured into a dialysis bag, which was dipped in 250 ml of PBS as a release medium. The obtained release medium was constantly stirred at 37 °C up to 168 h. At each time point (1, 2, 3, 24, 41, 92, 144, and 168 h), 4 ml of the release medium was removed, and the absorption value at the wavelength of 279 nm was determined by a UV-visible spectrophotometer. Thymol concentration was calculated based on the prepared absorption-concentration standard curve, as previously mentioned in the section of EE of thymol. The same volume of the fresh PBS was added to the release medium. Similarly, the thymol release profile from the prepared film was calculated by UV-visible spectrophotometry. In brief, 12 mg of silk film was dipped into 12 ml of PBS at 37 °C. At the altered time points (1, 2, 3, 24, 41, 92, 144, and 168 h), 3 ml of the release medium (PBS) was removed as a sample and replaced with 3 ml of a fresh PBS. The thymol concentration in the removed sample was assessed using a UV-visible spectrophotometer at the wavelength of 279 nm.

***Cytotoxicity and cellular morphology assessments***

The cellular viability of the fibroin films without thymol and with both free and niosomal thymol was evaluated using L929 fibroblasts (Cell Bank, Pasteur Institute, Iran). The tissue culture plate was considered as a control sample. The cells were cultured in RPMI medium (Gibco, USA) supplemented with 100 µg/ml of streptomycin, 100 µg/ml of penicillin, and 10% fetal bovine serum (Gibco). The UV-sterilized samples were placed in 96-well plates, and 4 × 10^4^ of the cells were seeded on the films. The cytocompatibility evaluation was carried out using MTT (Sigma) colorimetric analysis at 24, 48, and 72 h. Briefly, after discarding the culture medium, 100 µl of MTT solution (5 mg/ml in PBS) was added to each well of the plate. The samples were incubated at 37 °C for 4 h, and the medium was then replaced with dimethyl sulfoxide solution (Sigma) to dissolve formazan crystals. A microplate reader (ELISA reader, URIT-660, China) was used to calculate the optical absorbance at 570 nm. The morphology of the cells cultured on the films was observed by SEM imaging. After culturing for 72 h, the cells were fixed with 2.5% glutaraldehyde (Sigma) for 1 h, followed by constant dehydration using a serial dilution of ethanol (10, 30, 70, 90, and 100% v/v) and gold sputter coating for SEM analysis.

***In vitro antibacterial activity assay***

The agar diffusion assay was used to evaluate the antimicrobial activities of SF, SF/Th, and SF/N/Th samples^[^^29^^]^. *E. coli* (ATCC25922), *S. aureus* (ATCC 25923), and *P. aeruginosa* (ATCC 27853) were supplied from the archive of Pasteur Institute of Iran. Bacterial suspension was prepared using the physiological serum, where the OD 625 nm equal to 0.1 represents bacterial concentration of 1.5 × 10^8^ CFU/ml. The obtained suspension was then spread on the Muller-Hinton agar medium (Merck). The sterilized samples (10-mm diameter) were located on the agar plate and incubated at 37 °C for 24 h. Finally, the inhibition zone of the samples was investigated.

**Statistical analysis**

Statistical analysis of the data from all the characterizations was conducted using one-way analysis of variance (ANOVA) for one independent factor. For two independent factors, two-way ANOVA using SPSS 22.0 software was used. Each value was reported as mean ± SD with three replications, and the *p* < 0.05 was considered as statistically significant. 

## RESULTS AND DISCUSSION

Due to the excellent antibacterial properties of thymol as a plant-based natural drug, it has the potential to be used extensively for preventing infection in implant site^[^^30^^]^. Stability and bioavailability of thymol could be improved by nanocarriers such as niosomes. This study was focused on the synthesis and evaluation of the thymol-loaded niosomes incorporated into the fibroin film for preventing infection in the implanted sites.

**Morphology and size of niosome**

**Fig. 1:**
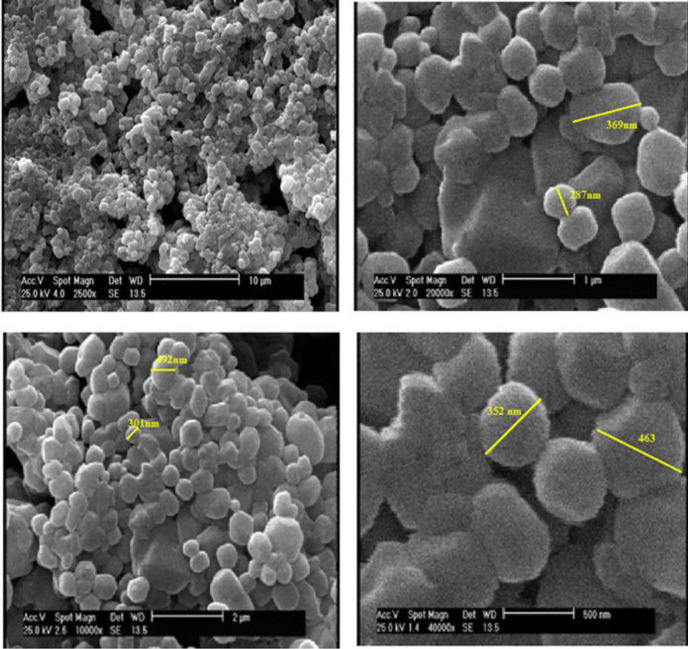
SEM images of the synthesized niosomes

 The morphology and shape of niosome were obtained by SEM images (Fig. 1). Vesicle size values were estimated in the range of 250-600 nm (mean = 321.254 ± 1.4 nm) using Image Analysis software (Image J, 1.51, Wayne Rasband). The surface morphology of the niosome was spherical and disc-like^[^^31^^,^^32^^]^. Since the particles underwent freeze-drying for imaging, the estimated size would be marginally smaller than their actual size^[^^33^^]^. Therefore, DLS analysis was used to assess the size distribution of the synthesized niosomes in suspension state. Z-average size and polydispersity index were determined around 395.87 ± 2.1 nm and 0.50093, respectively, which is in good accordance with an earlier study^[^^31^^]^. DLS estimates hydrodynamic diameter; therefore, the larger diameter in SEM analysis shows the smaller size. 

**SEM image of niosomal films**

**Fig. 2:**
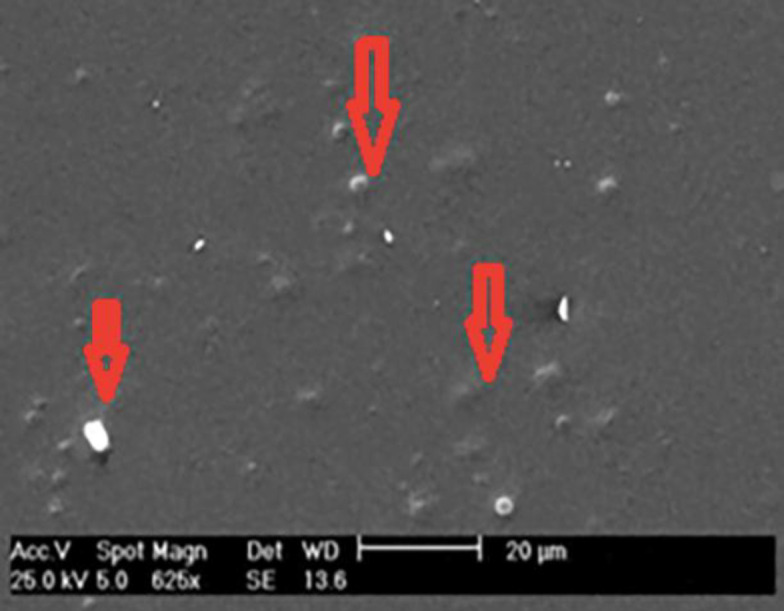
SEM image of the niosome-loaded fibroin film. The presence of niosome on the surface and inside of the film is shown by an arrow

 To investigate the presence of niosome and its distribution on the silk film, the SEM analysis was used. As shown in Figure 2, niosomes were dispersed uniformly inside and on the surface of the film. The uniform distribution of niosome can help the sustain releasing of the antibacterial drugs from the implant, supporting the long-term antibacterial effect. Our release study showed promising results, although further analysis is required to prove this hypothesis. 

**In vitro** **release study**

***Encapsulation efficiency***

Thymol was successfully incorporated into the niosome with 81% EE, which was a suitable encapsulation rate compared to that reported in the other studies^[^^34^^-^^35^^]^. Sohrabi et al.^[^^35^^]^ reported 73% efficiency for drug entrapment in niosomal carrier for moxifloxacin as a antimicrobial agent. Tavano et al.^[^^34^^]^ encapsulated tetracycline hydrochloride in the niosomal nanocarrier with EE of 80%. The high EE of the thymol can be related to the large capacity of the lipophilic moiety of the span 60 bilayers due to having long alkyl chains^[^^36^^]^.

***Standard curve for thymol***

**Fig. 3:**
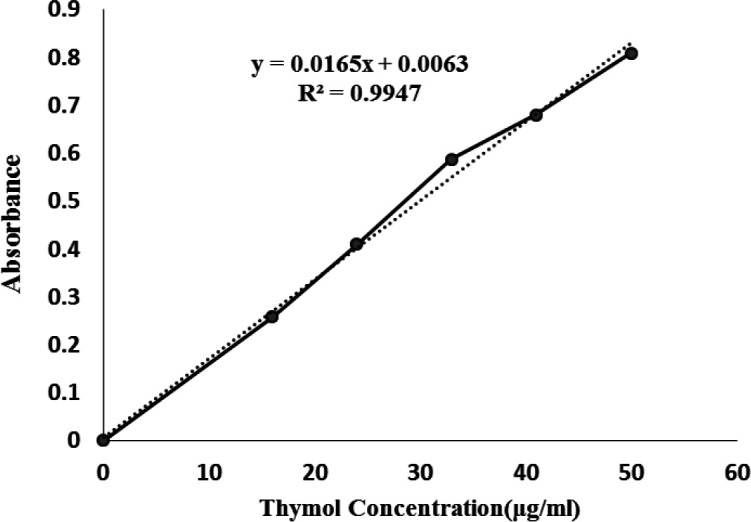
Standard curve (absorbance/concentration) prepared for thymol

Thymol standard curve was plotted by using UV-visible spectroscopy. The absorbance of solutions with different concentrations (0, 16, 24, 33, 41, and 50 µg/ml) was measured at 279 nm (Fig. 3). The fitted equation was used to measure thymol concentration.

***In vitro release analysis***

**Fig. 4:**
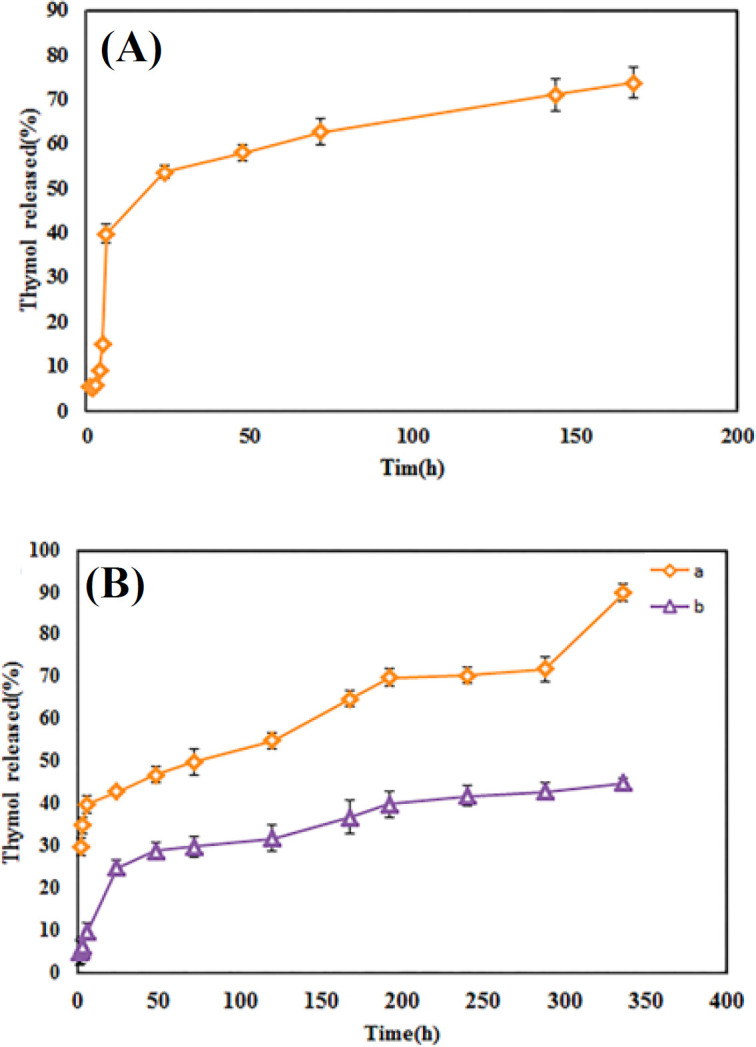
In vitro release profile of thymol from (A) niosome and (B) fibroin films containing (a) thymol and (b) niosomal thymol

The thymol release pattern from niosomes was examined at the physiological simulated environments (37 °C and pH 7.4). As shown in Figure 4A, the release behavior has a biphasic profile. The burst release was observed in the initial phase (0 to 6 h) due to the possible diffusion of drug molecules adsorbed to the surface of niosomes. During the second phase (6-168 h), the slower release rate was provided through the diffusion of the entrapped drug molecules out of the vesicles. Such release profile has also been shown in previous studies. Jin et al.^[^^37^^]^ showed a sustained pattern of release in all media via a biphasic profile. However, decreasing the pH resulted in the lower burst release. Given the thymol antimicrobial application, its initial burst release can prevent the early colonization of bacteria, while the slower release after 24 h may maintain the antimicrobial properties for a longer time^[^^19^^]^. Figure 4B shows the release behavior of thymol from the SF/Th and SF/N/Th films. No burst release was observed in the niosomal film sample in contrast to SF/Th films. After 14 days, the release of thymol from SF/Th and SF/N/Th reached almost 90% and 35%, respectively. As it seems, the niosomal film showed an extended release compared to the film containing free drug. Therefore, niosome caused a delay in the thymol release from films, which can be beneficial for the prevention of infection during the healing process via maintaining the level of thymol concentration in the implant site. However, SF/Th films had faster release, which might not protect the implant site during the healing.

**Cytotoxicity and morphology assessments**

**Fig. 5:**
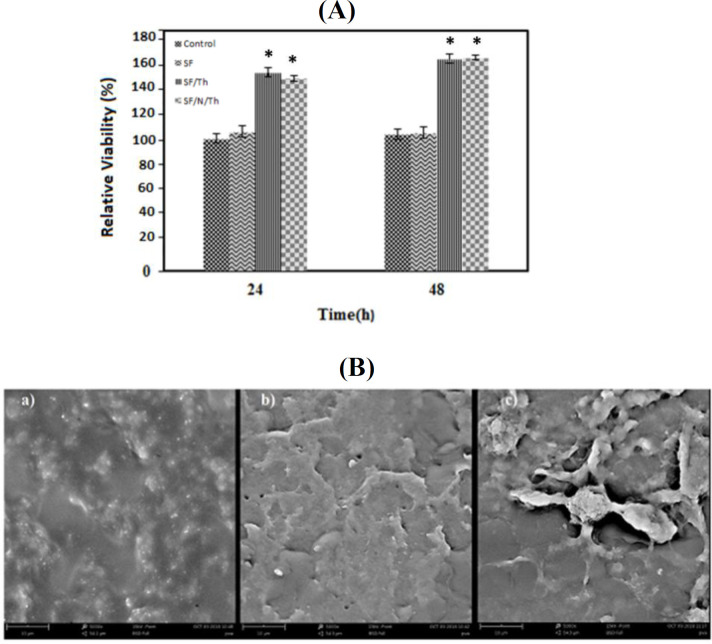
*MTT assay results for determination of L929 fibroblast cytocompatibility of the scaffolds* *(* *A). Data were shown as mean * *± SD for three independent tests.* *Asterisks indicate a statistically significant difference (*p* < 0.5); (B) SEM images of the fibroblast cells cultured on (a) SF, (b) SF/Th, and (c) SF/N/Th films after 72 h. Cells show almost confluent spreading on the surface of the films*

The MTT assay was used to evaluate cell viability after 24 and 48 h (Fig. 5A). As it can be understood from the Figure, there was no significant difference in optical absorbance between the control and SF samples (*p* > 0.5). However, SF/Th and SF/N/Th films showed higher cell viability compared to the control and SF samples (*p* < 0.5), suggesting that thymol has no toxicity on the cell viability. Cell morphology after 2 h is shown in Figure 4B in which the fibroblast cells were well-flattened and spread on the surface of the films and covered the film surface. Also, the SF/N/Th exhibited better cell attachment and spreading compared to SF/Th and SF. These results highlight the potential of adding thymol with/without niosome to the implant to improve the cell viability and attachment without causing any toxicity^[^^33^^,^^38^^-^^41^^]^. 

**Antibacterial assay**

**Fig. 6:**
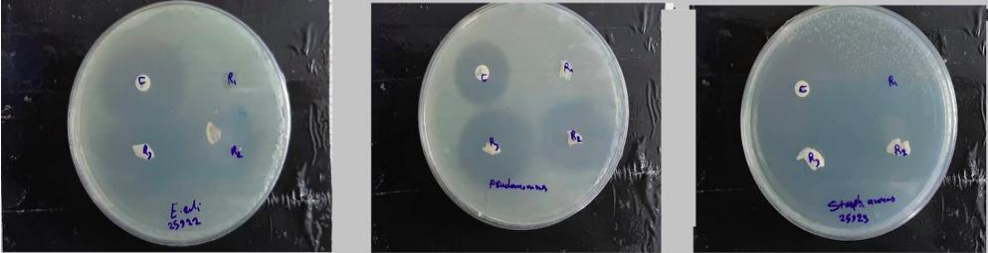
Inhibition zone of *S. aureus, E. coli*, and* P. aeruginosa* around films after 24 h of incubation in a Muller Hinton agar plate. C, positive control (ciprofloxacin); R_1_: SF; R_2_: SF/Th; R_3_: SF/N/Th

The antibacterial assay of SF, SF/Th, and SF/N/Th against* E. coli*,* P. aeruginosa*, and* S. aureus* are shown in Figure 6. SF/Th and SF/N/Th films exhibited antibacterial activity against both Gram-negative and Gram-positive bacteria, but SF/Th film showed greater inhibition zone because of its faster thymol release compared to the niosomal sample in the initial time. Therefore, it is expected that during the time of release, the antibacterial activity of SF/N/Th will be increased. Also, more significant inhibition zone was observed in *S. aureus* samples, indicating the higher antibacterial properties of thymol against Gram-positive bacteria. Such antibacterial activity of thymol can be related to its limited solubility because and its lipophilic nature. Such characteristics cause better interaction with lipidic cell membranes of bacteria and damage its integrity. Thymol can also bind to the genomic DNA and cause a change in its secondary structure and morphology. The lower activity of thymol against *E. coli* bacteria can be attributed to the hydrophilic properties of their complex cell wall due to the outer polysaccharide surface that can supply the defense against the highly hydrophobic thymol effects^[^^42^^-^^44^^]^. The inhibition zones around SF/Th and SF/N/Th were observed about 17 mm for *Staphylococcus aureus*, 16 mm for *E. coli*, and 15 mm for *P. aeruginosa*. As Gram-positive bacteria (particularly *staphylococcus aureus*) are mainly associated with high infection at the implant site^[^^45^^]^, using niosomal thymol-incorporated implants seems to eliminate and inhibit possible infection and reduce the chance of implant failure due to persistent infection.

In the present study, thymol-loaded niosomes were successfully prepared and combined with the SF films for the controlled release and local delivery of thymol to prevent and treat the infection in the implant site. The study of thymol release from the niosomal film showed a sustained manner over 14 days. Also, in vitro biocompatibility analysis of SF/Th and SF/N/Th exhibited a high viability and adhesion of fibroblasts. The antibacterial assay of the SF/Th and SF/N/Th scaffold demonstrated satisfactory effect against both Gram-negative and Gram-positive bacteria. Overall, the SF/N/Th can be introduced as a promising biomaterial for preventing infection in the implant site. 

## DECLARATIONS

### Acknowledgments

The authors would like to thank the research staff of “Advanced Scaffold Lab” for their kind helps and supports.

### Ethical statement

All the authors have read and approved the contents of the final manuscript and agreed to publicize this manuscript

### Data availability

 Data supporting this article are included within the article and supplementary file.

### Author contributions

RN: data collection, data analysis, and manuscript writing; RI: project development, data management, manuscript editing; MB: data collection and manuscript writing; SN: manuscript writing/editing. All authors have given approval to the final version of the manuscript.

### Conflict of interest

None declared.

### Funding/support

There is no funding supported this project.
